# Dental caries: A complete changeover, PART III: Changeover in the treatment decisions and treatments

**DOI:** 10.4103/0972-0707.73383

**Published:** 2010

**Authors:** Usha Carounanidy, R Sathyanarayanan

**Affiliations:** Department of Dentistry, Pondicherry Institute of Medical Sciences, Pondicherry, Karnataka, India; 1 Department of Conservative Dentistry and Endodontics, Bapuji Dental College and Hospital, Davangere, Karnataka, India

**Keywords:** Antimicrobials, caries vaccine, chlorhexidine, CPP-ACP, fissure sealant, fluoride, minimal intervention, nonoperative treatment, operative treatment, prevention, probiotic, remineralization, restoration, slow fluoride releasing device, treatment decision

## Abstract

Comprehensive management of dental caries should involve the management of disease as well as the lesion. Current decision making process in cariology is influenced by numerous factors such as the size/ depth/ activity of the carious lesion and age/ the caries risk status of the patient. Treatment decisions should involve planning the non-operative/ preventive treatment for non-cavitated or early cavitated lesions and also formulating operative treatment for cavitated lesions. Apart from these two responsibilities, a clinician should also be knowledgeable enough to decide when not to interfere in the caries dynamics and how frequently to recall the patient for follow-ups. The non-operative treatment prescriptions vary in dose, intensity and mode of delivery according to the caries risk status. Minimal invasion and maximal conservation of tooth structure has become the essence of current operative treatments. This part of the series elaborates on the paradigm shift in the management of dental caries.

## INTRODUCTION

A brief recapitulation of the previous part on diagnosis and detection[1] will provide a scaffold for further discussion.

1. Lack of definite delineation in the physiology-pathology continuum of the caries process poses challenges in the diagnosis and detection of dental caries.
2. Lesion detection primarily focuses on the detection of very early demineralizations that are not easily discernible.
3. Refined visual –tactile criteria in conjunction with sophisticated gadgets are used to reliably detect the earliest signs of initial demineralization. Once detected, the lesions are further assessed for extent and activity.
4. The ‘risk model’ aspect of the caries risk assessment not only helps to categorize the risk status of patient as high/ low/ moderate, but also helps to diagnose the caries disease. It aids in identifying the dominant biological determinant that is responsible for the disease.
5. The prognosis of the disease and the rate of progression of the lesion are assessed by the ‘predictor model’ facet of caries risk assessment.

The crux of the exhaustive process of the diagnosis of any disease is its control and cure. The treatment decisions for any disease usually focus on the management of the signs, the symptoms and the elimination of the cause/s of the disease. Additional decisions are also taken to prevent the recurrence of the disease. Most often, elimination of the causes alone may result in the inadvertent reversal of the disease signals. But for certain diseases, the treatment decisions bifurcate to manage the manifestations or the ravages of the disease separately. Dental caries is such a disease, where the management is dichotomous, targeting the disease and the lesion. Caries disease is managed by background level treatments directed against the dominant causative factor/s. These treatments are mostly non-invasive in nature. Carious lesion management addresses the demineralization and remineralization cycle at the tooth mineral level, by using both non-invasive and invasive treatment strategies.

## TREATMENT DECISIONS – THEN AND NOW

Treatment decision was deceptively simple when dental caries was equated to just a cavity in the tooth and treatment was equated to just filling the cavity. The intense focus was on the ‘art’ of creating a good restoration. Material science advancement and technical revolution in high speed cutting gadgets, though improved the quality of the restorative treatment, ironically sidelined the disease nature of dental caries. For centuries, this mechanical solution for a biological problem prevailed.

It is not that the profession was ignorant of the biological nature of dental caries. GV Black in 1908 in his text book Operative Dentistry stated that this attitude is “….*an anomaly in science that should not continue. It has the apparent tendency to make dentists mechanics only*.”[2] In 1967 Massler stated that “…*disturbing*….*to witness the overly focused attention of some dentists upon the operative and restorative phases of dentistry, the drilling and filling of teeth, to the neglect of the disease process which causes the lesion (cariology) and the pre operative treatment of the wounded tooth-bone*.”[3] The concept of prevention of dental caries is not new either. Since McKay stumbled upon the ‘*Colorado brown stain*’, fluorides’ anti-cariogenicity has been well studied and proved. Introduction of water fluoridation kick started a plethora of preventive concepts. But it is disturbing to note that prevention at the individual patient/ tooth/ surface level still receives less attention in practice, compared to population/ community level. Even if preventive managements are included in the individual treatment plan, they generally are added as supplementary to the restorative treatment, not as the primary treatments. Young *et al*. criticizes that the term prevention has become a term that has been “*blanched and simplified into to only mean ‘brush and floss’ and ‘don’t eat sugar*’”.[4] Kidd *et al*. astutely observed that the oral prophylaxis or dietary counseling or oral hygienic instructions are given by the dental assistants, not by the dentist, thus making the patients think that they are unimportant.[5] Low patient compliance is the direct result of this wrong message. Perhaps, prescriptions and advices fade out in the face of the action packed filling procedure!

Out of innumerable reasons that can be incriminated for such unjustifiable protocols, the prime accused is the educational system. A dental graduate is still trained to ‘do’ the ‘things right’ (*e.g. getting the cavity and restoration perfectly*) and not trained to ‘think’ the ‘right thing’ (*e.g. is a restoration really needed*). Pass or fail is still determined by the quality of his/ her restoration. Despite a common objective of treating caries, both restorative dentistry and preventive dentistry do not converge in academics as well as in practice. They always remain as parallel entities failing to impart a wholesome knowledge for a comprehensive treatment plan.

However, with the current changeover in all dimensions of dental caries, productive and desirable changes are evident in the management decisions. First, there is now an intense focus on the preventive strategies and minimal/ non-invasive management of the incipient lesions. Essentially, all the treatment strategies under the umbrella of prevention either alter or modify the causative factors in the dental caries etiology, such as diet factor/ host factor/ salivary factor and the microbial factor. Thus, their role in caries disease management is very crucial. These strategies are categorized as primary, secondary and tertiary.[6] Primary prevention prevents the onset of new lesions in the healthy tooth and secondary prevention controls or arrests the progress of already existing incipient demineralization. Thus, it can be understood that preventive treatment not only just prevents the disease, but also treats the lesions. To underscore the importance of the preventive protocols as disease management solutions and also to emphasize that they are as equally important as the restorative component of the treatment decision, a change in the nomenclature has been suggested. Prevention is now preferred to be called as *non-operative treatment*,[5] thus giving it an equal status as *operative treatment* or restorations.

Second, the prime objective for the operative treatment or the restorative treatment has been shifted to plaque control. Generally, over the years, operative treatment has shrunk in the size as well as in the indications. Possibility to detect the carious lesion at the non-cavitated stage and the possibility to remineralize/ arrest the lesion even in the initial cavitated stage have considerably reduced the clinical situations that necessitate restorations. It has been suggested that the cavitated smooth surface caries and the root caries can be handled without restorations just by enhancing plaque control in these areas. The undermined enamel needs to be removed and the surface should be finished and polished. Cleaned twice with fluoridated paste, the active lesions in these areas have been reported to be converted to inactive lesion.[7]

Though with adhesive restorations, the profession has traded off the concept of longevity of restorations for esthetics, this technology indeed has to be credited for paving the way for minimal invasive dentistry. The advancements in this field coupled with biological re incarnation of cariology have enabled the profession to do away with many mechanical principles of cavity preparation that magnified the size of the cavity. (For instance, ‘extension for prevention’)

A treatment decision tree for a carious lesion shall emerge as the following questions are answered:

1. Is there a need to intervene for this lesion?
2. If yes, does it require a non-operative treatment or an operative treatment?
3. If preventive treatment, what is the regime?
4. If restorative treatment, how minimally can it be done?
5. Does the patient fall under high/ low/moderate risk status?
6. Which is the dominant causal factor/s?

**Figure 1:**
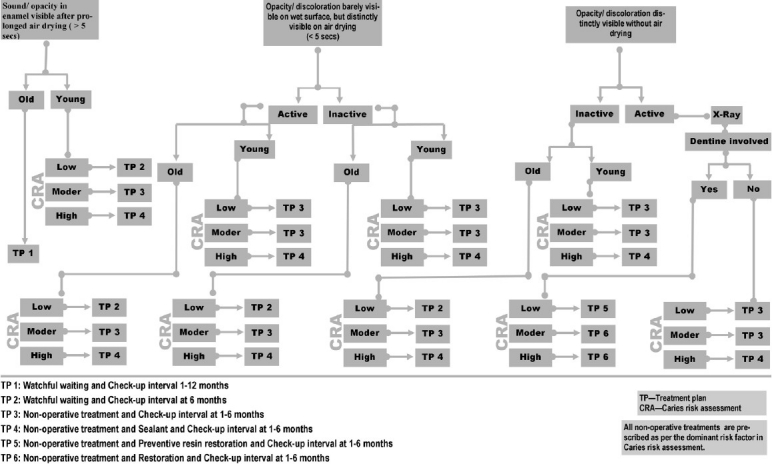
Treatment decision tree for a non cavitated pit and fissure lesion. (Adapted and modified)[8] The treatment decision is influenced by the activity of the lesion, the risk status and the age of the patient. The decisions include non-operative treatment, operative treatment and follow-up period

Figure 1 depicts a classical treatment decision tree for a pit and fissure lesion.[8] On close study, three factors emerge that critically influence the treatment plan, especially for non-cavitated lesion. They are lesion activity, age, and caries risk status.

Color and texture of the lesion can differentiate an active lesion from an inactive lesion. Actively progressing lesions definitely have to be interrupted in their dynamics, whereas the inactive lesions, even if they are in later stages of demineralization need not be disturbed. They are kept under watchful surveillance. In addition, treatment decision for a similar lesion can vary between a young tooth and an old tooth. In a susceptible age group, there is more likely a chance for a seemingly inactive non-cavitated lesion to transform into an active lesion. Therefore, a more intense therapy is advocated for young tooth compared to old tooth.

The most important factor that can modify the clinical judgment in management is the caries risk status of the patient. Caries risk assessment evaluates the risk status of the patient and also identifies the dominant disease causing factor/s. Once they are identified, specific tailor-made treatment is planned targeting them. In the absence of this identification, the non-operative prescriptions tend to be either insufficient or unnecessary. Assessment of the risk status also enables the prognostication of the activity of lesion. For example, an inactive lesion in a high risk status shall receive an intense treatment than in a low risk status.

**Figure 2:**
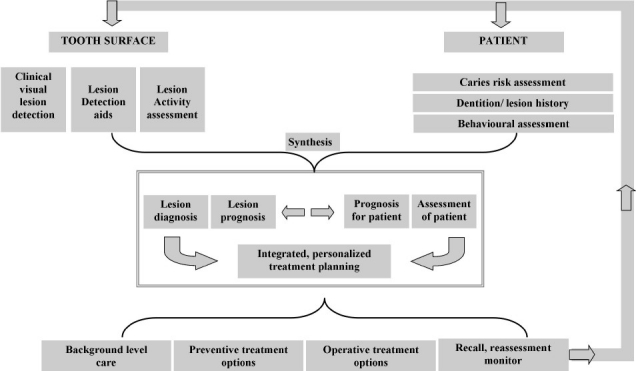
Contemporary ICDAS-enabled, patient-centered caries management framework. The treatment decision is influenced by the nature of the lesion and risk status of the patient[9]

Exhaustive guidelines have been laid down for both non-operative and operative treatment strategies based on caries risk assessment. One such assessment is the CAMBRA (Caries management by risk assessment) guideline.[4] This lays out a complete guideline with regard to when to take a bite-wing radiograph, when to recall the patient, when to do the caries risk assessment, whether to prescribe minerals / antimicrobials, how much to prescribe and for how long. This exhaustive guideline based on evidence is given for all the caries risk status. It also includes the indication for pit and fissure sealant procedure. ICDAS (International caries detection and assessment system) guidelines are also available.[6] Figure 2 depicts the newer philosophy proposed by ICDAS for caries management that takes into consideration lesion status and the risk status.[9] It should be understood from these guidelines, that, be it invasive or non-invasive treatment or be it cavitated or non-cavitated lesion, the intensity/ severity/ frequency of all types of treatments vary with caries activity, age, risk status. The decisions to ‘wait and watch’ and the period of follow-ups also form important part of treatment decision. ‘Watchful wait’ is a prudent non-operative strategy, and should not end up as supervised neglect. The frequency of recall visits and the actions to be taken in each recall visit will again depend on the risk status of the patient and activity status of the lesion.

## NON-OPERATIVE TREATMENT – THEN AND NOW

Non-operative treatments act at various planes of the dental caries disease dynamics. It should be borne in mind that no single modality can effectively control the disease, as the disease process is a complex interaction of the multiple causal determinants. Various treatment modalities have to act in concert to combat the disease comprehensively.

### Treatment options that act at the tooth level – Then and now

Discovery of fluorides’ anti-caries effect borders serendipity. Lack of caries affliction in fluorosed teeth aroused the curiosity of the profession, precipitating a series of scientific explorations into this chemical, for its anti-caries effect. Many years of evaluation has culminated in very strong evidence on the remineralizing capacity and also in its capacity to increase the tooth’s resistance to demineralization. Formation of fluorapatite crystals that are more acid resistant than hydroxyapatite crystals has been attributed as the primary mechanism of action.[10] However, prevalence of dental caries even after a widespread systemic fluoridation raised queries on the pre-eruptive effect of fluoride on enamel. Owing to this lacuna, post-eruptive effect of fluoride became the topic of interest. Presence of free fluoride ions in the biofilm and the oral fluid is now considered to be more important than the concentration of bound fluoride in the enamel. It has been found that, under an acid attack, it is the free fluoride ions, along with the calcium ions, that influence the remineralization process.[11] Thus emerged the wide spread use of topical fluorides, where free fluoride ions were made available at the tooth’s vicinity in high concentration.

Topical fluorides provided versatility in mode of application, control of dosage and more significantly, allowed individualized fluoride regime based on caries activity and risk. They have been reviewed systematically for their efficacy in caries prevention either alone or in combination.[12–17] Among all the topical applications, fluoridated dentifrice along with systemic water fluoridation is considered as the preferred mode of administration.[18] Other topical applications usually are intended to supplement this combination, especially in high risk individuals.[19] If dental caries is a continuous dynamic process, then control or prevention should also be a constant and continuous effort. Thus instead of increasing the frequency of application, the retention rate and the sustained-release effect became important requisites for prolonging the action of fluoride. Gel and varnish as the modes of delivery provide better retention of fluoride over the enamel. Highest level of evidence is available on the superior anti-caries effect of both delivery methods.[20]

Inspired by glass ionomer cement’s fluoride releasing capacity, other restorative materials were developed with the same virtue. But evidence is lacking regarding their effective fluoride release.[21] Sustained and controlled release of fluoride is being tried by the use of slow-release fluoride devices.[22] These devices are attached to the tooth surface by means of an adhesive. The fluoride from these reservoirs is then slowly released to the surrounding. A copolymer membrane device is available that is a small pellet made of a fluoride containing copolymer matrix surrounded by a rate controlling copolymer membrane. When the matrix gets hydrated, the fluoride dissolves and saturates the matrix. Thus, the ions move from saturated matrix to less saturated membrane and then to the saliva. This device can release 0.02-1 mg F/day, for up to 180 days. Glass beads that dissolve over a period of time, releasing fluoride, have also been evaluated. These beads can store fluoride in concentrations from 13.3% to 21.9% and release it even up to two years. Mixture of hydroxyapatite and sodium fluoride is under evaluation as a slow-release device. Constant supply of fluoride that is not affected by the poor compliance of the patient is an advantage with this system. However, concern exists in the ability to retain these devices for a long time on the tooth surface.[23]

The fluoride uptake is influenced by concentration of calcium and phosphate ions in the saliva or biofilm. For every two fluoride ions, ten calcium ions and six phosphate ions are required to form one unit cell of fluorapatite (Ca_10_(PO_4_)_6_F_2_). Thus, topical applications of calcium and phosphate complexes are being used to enhance fluoride remineralization. Although calcium and phosphate in soluble and insoluble forms have their own limitations, certain specific forms are available commercially to improve the bio availability of these ions. Unstabilized amorphous calcium phosphate (ACP), casein phosphopeptide stabilized amorphous calcium phosphate (CPP-ACP) and bioactive glass containing calcium sodium phosphosilicate are some of these systems available.[24] CPP-ACP is available in dentifrice formulation, as a mouth rinse and as a non-sugar containing chewing gum. They are also available in combination with fluoride. The minerals are released under an acid challenge to supersaturate the saliva and help in remineralization. They may be an important and effective adjunct to fluoride topical therapy in treatment of incipient lesions. But insufficient clinical trial reports are available to make a strong recommendation for this mineral combination.[25]

Laser-activated fluoride (LAF) is a method by which the resistance of the enamel to demineralization is increased by using a combination of laser irradiation and topical fluoride application. Laser irradiation has been found to reduce the critical pH of dissolution of hydroxyapatite crystals from 5.5 to 4.31. In the presence of fluoride, further reduction has been observed. However, the evidence is only at the *In vitro* level.[26]

### Treatments that act at the microbial level – Then and now

The effective use of minerals in the fortification of the tooth against acid attack shall go futile, unless the micro organisms that produce the acid are dealt with. Furthermore, a thick biofilm on the tooth surface prevents active penetration of fluorides into the enamel. Elimination of the entire biofilm from the tooth surface, either mechanically or chemically, is not only impossible but also imprudent. Plaque biofilm is an inherent part of the host conferring a certain degree of defense to the tooth from foreign invasion. However, mechanical means such as brushing and flossing have not only been effective in reducing the plaque mass, but the efficacy of fluoride is also enhanced in reduced plaque thickness.

Mechanical blocking of the susceptible tooth sites by sealants, deprive the favorite nidus for growth of cariogenic organisms. Pit and fissure sealant as a primary or a secondary preventive measure has been in popular use since a long time and has enough evidence to back-up its continued use.[27] Sealing the proximal initial lesions with adhesive resin has been evaluated recently and found to be effective in retarding the lesion progression, if not arresting the progression.[28]

It is a logical thinking to use antibiotics and antiseptics for dental caries, similar to any bacterial induced disease. But the organisms causing caries are innate part of the host micro biota, not foreign organisms. Therefore, any anti microbial treatment should selectively target only those cariogenic organisms without harming the others in the colony. Indiscriminate use of an antimicrobial with a broad spectrum, over a long period, can alter the character of the entire bacterial community resulting in the emergence of new resistant strains. With the background understanding of the ecological plaque hypothesis, the current objective is to change the biofilm ecology from cariogenic to non-cariogenic.[29] Furthermore, there is a very weak level of evidence for the caries preventive effect of even the widely used medicament such as chlorhexidine gluconate. Although this medicament is effective in reducing mutans streptococci count, there has been no correlation detected between this effect and its caries inhibiting effect in clinical studies.[30] Weighing its established side effects and the inconclusive evidence for benefits, the clinical usage of chlorhexidine is even being dissuaded.[31] However, ‘inconclusive’ evidence does not necessarily mean ‘not effective’;[30] therefore, the use of antimicrobials, in the current times, has been restricted to caries-active individuals and to high caries risk individuals. Instead of prescribing it as a routine medication, it is prescribed as a regime for a short duration for a high risk individual, only if the risk assessment points toward a dominant microbial cause.[32]

Instead of using chemicals, the micro organism can be countered with another micro organism, as the old Indian proverb goes; ‘use a thorn to remove a thorn.’ This is the concept behind the *replacement therapy* or the *probiotic therapy*. The disease causing ‘*wild strains*’ of bacteria can be displaced from their ecological niche, by introducing genetically modified form of the same organism, called as the ‘*effector strain*’. These effector strains saturate these niches and prevent the colonization and multiplication of the wild strains again. A common example for a probiotic approach is the use of lactobacilli in intestinal infections. The mutans streptococci are genetically re-engineered to form strains that lack the inherent pathogenicity. One such strain developed lacks the enzyme lactate dehydrogenase, and so is incapable of producing lactic acid responsible for demineralization. Yet another genetically engineered streptococci that produces alkali instead of acid in the biofilm metabolism is under evaluation.[32,33]

Almost 50 decades have past in the pursuit of caries vaccine. Still none is available for effective clinical usage. Active immunization had been the prime line of research since the definite causal relationship of streptococcus mutans was established. Although two serotypes of this species were identified as s. mutans and s. sobrinus, the focus has been only on the antigenic property of the s. mutans. Study of molecular pathogenesis of this organism gave clues as to its primary antigenic features. The adhesion and proliferation of the organism is facilitated by three factors. A surface fibrillar adhesin enables adhesion of the micro organism to the salivary pellicle on the tooth. The bacterial enzyme glucosyltransferases convert the dietary sugars into water insoluble/ water soluble glucans, which is a sticky polysaccharide. Glucan binding proteins on the cell surface of the bacteria further enhance adhesion and proliferation on the tooth surface. These three antigenic factors have been exploited in an active vaccine production.[34]

Focus has shifted from stimulating the IgM, and IgA antibodies, to stimulating the salivary SIgA, which is thought to provide better immunity to the tooth structure. Thus, mucosal immunity has become the current platform for caries vaccine and vaccine administration has shifted to nasal administration.[35]

As any vaccine should be instituted before introduction of the infectious agent into the system, caries vaccine should also be administered in the pediatric stage. A period of ‘window of infectivity’ has been observed in the bacterial colonization of infants’ oral cavity. From the middle of the second year to the end of the third year is the period when colonization occurs and the source is most likely the feeding mother. Thus, it is logical to administer the vaccine before this period.[36] Various pre clinical studies have established the effect of these vaccines, but clinical trials in humans are still pending verdict. Although active immunization can confer long-term immunity, it is not without adverse effects. The probability of cross-reactivity to the heart tissue has not been ruled out yet.[37] The risk is more compared to the benefits; especially for a non-life threatening disease that can be effectively controlled by other safe preventive measures. Doubts linger about the comprehensive efficacy of a vaccine that targets only streptococcus mutans without taking into consideration the interaction or the role of other causal microbes, such as streptococcus sobrinus.[38] Coupled with the cost in manufacturing and the complexity of clinical trials, the above mentioned reasons have been incriminated as reasons for the retarded growth of active vaccine.

To cut across these hurdles, passive immunization is currently being adopted in caries immunization.[39] Instead of actively producing antibodies in the host system, ready made antibodies to a specific antigen, produced elsewhere, is administered locally to obtain the desired result. The antigen, when administered to a cow or a hen, stimulates the production of antibodies that are secreted in the milk and in the egg yolk, respectively. Recombinant monoclonal antibodies are also being used in passive immunization. All have shown a short-term reduction in the colonization of s. mutans in the rats and monkeys. Lately, the transgenic antibody from plants (tobacco and potato), also called as the ‘*plantibody*’, has been manufactured and is under a clinical trial. These antibodies are applied locally to the tooth, mostly in the form of mouth rinse. However, only a transient drop in the microbial count lasting only for few hours does not satisfy the requirement of a vaccine, which is to provide a sustained availability of antibodies. This form of passive immunization is akin to any other mouth rinse, where lack of substantivity and retention might reduce the efficacy of the antibodies, necessitating frequent applications.[35]

### Treatments that act the salivary level - Then and now

One-stop solution to combat a salivary problem is to increase the salivary flow. Increasing the flow, improves the buffering capacity as well as the cleansing action. Not much has changed in this realization from yester years. Topical/ local therapies and systemic therapies are continued to stimulate the salivary flow.[40] Chewing gums are in the lime light now. Flavored chewing gums result in increased salivary flow through gustatory stimulation. Chewing gums containing no sugar/ sugar substitutes have been recommended for routine prescription. Xylitol containing chewing gums have received much attention in the recent past. Xylitol is sugar alcohol which has exhibited a bacteriostatic effect on the s. mutans. The cariogenic bacteria do not ferment these sugars to acid, so they are popular as non-acidogenic sugars. However, the caries-preventing effect of these chewing gums seems to be based on stimulation of saliva rather than the antibacterial effect.[41] Minerals such as CPP-ACP and fluorides are also added to the xylitol containing gums to combine their beneficial effects.

### Treatments that act at the dietary level – Then and now

Dietary counselling is the only treatment modality available under this section. Counselling is the toughest part of the caries management as it requires complete frankness, co-operation and compliance from the patient’s side. Dental caries is not considered as grave as diabetes mellitus, which is another diet-influenced disease; thus the above-mentioned commitment is not easily earned by a dental clinician. Drastically curtailing the sugar intake is not a prudent advice. Instead, caloric or non-caloric sugar substitutes should be suggested. Certain countries have ‘tooth friendly’ logo in the commercial products.[42] Probably statutory warning like ‘eating sweets is injurious to dental health’ would improve the dental awareness among patients! Current literature does not have any evidence relating a systematic dietary counselling and reduction in caries incidence.

### Operative treatment – Then and now

Numerous ultra conservative tooth preparation procedures have long been introduced to replace the high speed cutting tool that is reputed to cut relentlessly. Air abrasion, chemical removal of caries, atraumatic restorative technique and lasers are some of these methods. The prime objective of these procedures or devices is to be selective in the removal of diseased tissue and preserve majority of the unaffected. Some of them are not entirely new to field; probably could not sustain themselves in the amalgam era, as they had to satisfy the macro-mechanical retention requirement for silver amalgam. These ultra conservative procedures have re-emerged as novel operative methods, thanks to adhesive dentistry. They all possess the virtues of being conservative, painless and of being able to produce a rough cavity that is conducive for an adhesive restoration. Despite these advantages and of being in the field for more than three decades, none of these procedures have completely replaced the high speed drills as routine clinical method.[43]

A systematic review on conservative cavity preparations such as tunnel preparation, proximal box only preparation and preventive resin restoration reveals that the tunnel preparations resulted in failures and early re restorations. Although this procedure has a noble objective of preserving the marginal ridge, technical difficulty in accessing the lesion through a restricted path has been attributed as the reason for failure. Therefore, this procedure is no more recommended for routine use. The proximal slot preparation fares slightly better than tunnel preparation, probably because of an improved accessibility. The preventive resin restoration, where cavitated occlusal dentinal lesions are restored with a resin composite and the contiguous non-cavitated fissures are sealed with pit and fissure sealant, has been found to be an effective conservative treatment.[44]

Widening or beveling the entry of the fissure for better visualization, detection and management has been suggested for many years. Recent literatures call them as fissurotomy, or ultra conservative preparations.[43] It is interesting to note that this is very similar to the old procedures that were called as prophylactic odontotomy and enameloplasty. The common objective among them is to widen/ make the fissure shallow and either to leave it self cleansable or to restore with adhesive resins. This technique is also called as *caries* biopsy[4] if it is mainly intended to detect the lesions at the depth of the fissure.

The manners in which the cavitated dentinal lesions are handled are also undergoing a paradigm shift. The efficacy of caries detector dyes in differentiating the infected and affected dentin has been questioned and is found to be an unreliable factor. Use of the dye has been reported to result in over cutting of the tooth structure.[45] DIAGNOdent has been suggested to do the same, but its reliability is again questioned.[46] To confine the cutting only to the soft dentin, without damaging the hard dentin, instead of a metallic bur, a polymer bur (smart prep, SS White) has been introduced, that has a tendency to become blunt when it contacts the healthy, hard dentin. Evidence available regarding this, bur is sparse and varied.[43]

Along side, the real need for such complete removal infected dentine is also being questioned. It is now suggested that it is not mandatory to remove all the infected dentin from the cavity in the best interest of safety of the pulp. Fissure sealant procedures over carious dentin, step-wise excavation method[43] where caries is intentionally left out in deep cavities, are some of the procedures that follow this style. This concept places unshakable faith in the ability of the restoration/ sealant to provide the ‘perfect seal’. Achieving a marginal seal that totally deprives the leakage of nutrition to the left-over bacteria is aptly described as ‘seal is the deal’.

Objectives such as conservation of health, prevention/ eradication of the disease constantly drive the minimal intervention treatment philosophy of dental caries. Optimism surges every time a novel concept or method emerges. But in this evidence based era, to sustain as effective management tool, they have to undergo long-term randomized control clinical trials. Therefore, despite being effective, in the absence of such trials, they might be branded as having inconclusive or weak evidence. Lack of strong evidence, in turn, does not warrant their effective end-usage namely patient service.

## DENTAL CARIES IN CLINICAL PRACTICE – THE FUTURE

A vital question to be addressed at the end of this three part series is “Is clinical practice updated with these back-to-back changes in dental caries concepts?” Unfortunately, there exists a big gulf between research, education and practice in this regard. Research in cariology is sky-rocketing, bringing out the hidden facts of this age-old disease, but the education and clinical practice is adopting them in a snail pace. Education should empower the profession with latest developments in cariology and also inspire ways to practice them for the ultimate benefit of the patients. But conflicting objectives in education have detached ‘theory’ from ‘practice’. By large, in clinical practice, dental caries is still being treated symptomatically, just like common cold. The comfort level that has been acquired by clinicians from long years of practicing ‘restorative’ dentistry is probably too ‘comfortable’, preventing them from addressing the new nuances of caries. Dental caries and common cold has one thing in common; not threatening to life. But unlike common cold that does not have a cure, dental caries has abundant option to be cured and eradicated. Not utilizing them to the best effect, is indeed unacceptable, because, after all, improving the quality of life is as grave a responsibility as saving the life itself.
